# Pain Management in Metastatic Bone Disease: A Literature Review

**DOI:** 10.7759/cureus.3286

**Published:** 2018-09-11

**Authors:** Imama Ahmad, Munis M Ahmed, Muhammad Farhan Ahsraf, Anika Naeem, Azka Tasleem, Moeed Ahmed, Muhammad S Farooqi

**Affiliations:** 1 Internal Medicine, King Edward Medical University, Mayo Hospital, Lahore, PAK; 2 Graduate, Allama Iqbal Medical College, Lahore, Pakistan, Lahore, PAK; 3 Internal Medicine, King Edward Medical University, Lahore, PAK

**Keywords:** cancer, metastatic bone disease, cancer care, cancer pain, pain management, bone metastasis

## Abstract

Cancer means an uncontrolled division of abnormal cells in the body. It is a leading cause of death today. Not only the disease itself but its complications are also adding to the increase in mortality rate. One of the major complications is the pain due to metastasis of cancer. Pain is a complex symptom which has physical, psychological, and emotional impacts that influence the daily activities as well as social life. Pain acts as an alarm sign, telling the body that something is wrong. Pain can manifest in a multitude fashion. Management of bone pain due to metastasis involves different modes with some specific treatments according to the type of primary cancer. Over the years various treatment modalities have been tried and tested to improve the pain management including the use of non-steroidal anti-inflammatory drugs (NSAIDs), opioids, bisphosphonates, tricyclic antidepressants, corticosteroids, growth factors and signaling molecules, ET-1 receptor antagonists, radiotherapy as well as surgical management. The topic of discussion will cover each one of these in detail.

## Introduction and background

Cancer means an uncontrolled division of abnormal cells in the body. It is a leading cause of death today and according to the “World Cancer Report 2014” on September 3, 2014, the incidence and mortality due to cancer are increasing with the higher incidence in China among all the Asians. This incidence has shown remarkable progress in the year 2012. The report predicts that global cancer cases will increase rapidly from 14 million in 2012 to 19 million in 2025 and to 24 million in 2035 [1].

Not only the disease itself but its complications also are adding to the increase in mortality rate. One of the major complications is the pain due to metastasis of cancer. Statistically, approximately 60%-90% of patients with advanced cancer have the complication of variable degrees of pain during their lifetime, of which almost 30% of patients have been suffering from persistent severe pain [2]. Bone cancer pain occurs in many patients of cancer and the reason is metastasis to bone which later on leads to invasion of the surrounding tissues which leads to signal transmission through pain fibers and thus the perception of pain occurs [3]. Two-thirds of patients with advanced cancer are prone to bone metastases. The most common organs that give the metastases to the bone are lung, breast, prostate, and ovaries [4].

Pain is a complex symptom which has physical, psychological, and emotional impacts that influence the daily activities as well as social life. Pain acts as an alarm sign, telling the body that something is wrong. Pain can manifest in a multitude fashion. Pain behaviors such as spontaneous pain, hyperalgesia, and allodynia are related to the release of neurochemicals such as substance P as well as c-Fos, and Dynorphin expression. Bone metastases are a frequent complication in patients with advanced cancer.

Over the years, various treatment modalities have been tried and tested to improve the protocols of pain management including methods such as bisphosphonates, chemotherapy, surgery, nerve block, adoptive tumor immunotherapy, and gene knockout. But the clinical treatment of cancer pain is yet to focus on the three-step program as established by the World Health Organization. According to the degree of pain, the patients will be given a non-steroidal anti-inflammatory drug (NSAID) (mild pain) and/or opioid therapy (moderate and severe pain). However, there are many patients suffering from resistant cancer pain along with other complications of the treatment such as “mirror pain,” morphine tolerance, and constipation, respiratory depression for opioid drugs, stomach ulcers and kidney toxicity for NSAIDs [5]. These side effects have limited the use of these drugs for a longer period of time [6]. Due to the fact that the molecular mechanisms of bone cancer pain have not been elucidated, and that the side effects and tolerability of clinically available drugs cannot be overcome, some 45% of patients with cancer accompanied by pain cannot be effectively controlled [7]. May it be the stretch on the periosteum or direct effect of the destructive lesions, bone metastases cause excruciating pain. Among patients with cancer, there is substantial heterogeneity in how and at what time of the day pain is perceived. Treatment of such metastatic cancer patients is not complete if the pain is not considered as the fifth vital sign and addressed the way it deserves. The advances in molecular mechanisms involved in the bone pain would help in the improvement of the treatment modalities for pain control.

## Review

Bone cancer pain is a chronic pain with complicated pathogenesis. Various studies over the years have shown that bone cancer pain may be due to substances produced by tumor cells and inflammatory cells, as well as sustained activation of osteoclasts and nerve compression and injury caused by tumor growth and invasion in adjacent tissues [8]. It can also be caused by local pressures exhibited caused by increasing tumor sizes.

Clinical analysis of bone metabolism in patients with bone metastases showed that tumor-induced bone destruction (osteolysis) is closely related to the occurrence of cancer pain. Osteoclastic activity is under the influence of tumor necrosis factor alpha (TNF alpha) and other cytokines that are secreted by the cancer cells. Then, bone-resorbing osteoclasts secrete protons and acidic enzymes that dissolve the bone. This acidic environment activates the nociceptors resulting in pain perception. The severity of pain depends on the number of neurochemical changes at the dorsal root ganglia in the spinal cord [9]. Primary sensory neurons located in the dorsal root ganglia can be divided into two general types: A-fiber and C-fiber in which A-β fibers conduct the non-noxious stimulation, whereas A-δ fibers and peptidergic C fibers are sensory neurons innervating the bone with different receptors to feel different stimulations. These receptors are transient receptor potential vanilloid 1, cold receptor (cold-and menthol-sensitive receptor), transient receptor potential melastatin 8, mechanically gated ion channel P2X3 receptor, endothelin (ET) receptor, and PG receptor. This noxious stimulation can be converted to electrochemical signaling by these receptors which are transmitted to the central nervous system (CNS) where the pain is actually perceived as a sensation. Also, another neurotransmitter named ET-1 also increases during bone metastasis.

The hyperalgesia induced by cancer involves central and peripheral sensitization [10]. During continuous peripheral stimulation, the sensitivity of ganglia and its neurons changes, and pain threshold lowers, resulting in hyperalgesia. For central sensitization, the mechanism explains that neurochemical changes in the spinal cord causes hypertrophy of astrocytes and increased expression of dynorphin and c-Fos which decreases the pain threshold [11]. Another cause of bone pain in metastasis is due to the pathological factors that follow the weakening of bones.

Medical management of metastatic bone pain

It involves different modes with some specifying treatments according to the type of primary cancer.

Non-steroidal Anti-inflammatory Drugs

Non-steroidal anti-inflammatory drugs since long are in use for the treatment of pain control for all the diseases. They also have an anti-inflammatory effect that makes them an ideal drug for the inflammation caused by certain cancer types during extensive tissue invasion and destruction. A meta-analysis of 25 randomized controlled trials related to the use of NSAIDs in cancer pain stated that although NSAIDs significantly reduced cancer-related pain above placebo, their role in the treatment of bone pain due to metastasis is still under consideration [12]. More recently, a Cochrane review was done for the use of NSAIDs in cancer pain in 42 clinical trials. They were tested alone as well as in combination with opioids. The main deal lacked the evidence of their superior efficacy or safety of one NSAID over the other [13]. The basic mechanism for this drug is to inhibit cyclooxygenase (COX) enzymes that are involved in the production of prostaglandins to regulate various cell functions including pain perception. In tumor cells, COX-2 has increased activity. Therefore reducing the activity of COX would inhibit the perception of pain as well. In support of this notion, acute administration of selective COX-2 inhibitors to rodents with cancer-induced bone pain was done and the experiment demonstrated attenuated (pain) behaviors, whereas chronic treatment reduced tumor burden as well, and osteoclast destruction in addition to producing significant pain relief [14]. The main drawback is that its effects are limited due to the short duration of action and lack of long-lasting effects.

Opioids

The second most commonly used drugs are opioids. It is one of the most effective and widely used drugs for cancer pain. Opioid drugs produce a long-lasting analgesic effect. Therefore more than 80% of patients with cancer need to use opioids to improve or control pain in some part of their lives. Analgesic effect of opioids is largely dependent on μ-receptor saturation and is thus influenced by the type and severity of the pain, prior exposure to opioids, and individual distribution of receptors. Major side effects of opioid drugs are physiological dependence, tolerance, addiction, sedation, constipation, nausea, vomiting, and respiratory depression that limit the further application. Clinicians can adjust the opioid analgesic effect by the following two aspects. First, individualized treatment based on pharmacogenomics studies of the cancer type makes the best analgesic effect possible with minimal adverse reactions. Second, pharmacodynamics and pharmacokinetics studies of the drug make us achieve the best performance of opioids with minimal dosages. As far as the side effects are concerned, various medications are used to decrease its side effects such as metoclopramide for nausea, laxatives for constipation, and methylphenidate for sedation. Opioid sensitization is also a major problem that leads to a decrease in the efficacy of the drug when used for prolonged periods of time. There are some receptors that actively take part in its desensitization. These are N-methyl-D-aspartate (NMDA) receptors. Prolonged opioid therapy may both contribute to an apparent decrease in analgesic efficacy, regardless of the progression of the pain. Thus, in some instances, treating increasing pain with increasing doses of the same opioid may be futile [15].

Bisphosphonates

The third most commonly used drug is bisphosphonate which is generally used to cure the hypercalcemic states in the body. These drugs improve the acidic microenvironment of the local tumor bone tissue, causing a decrease in the dissolution of bone and thus reduce the activation of acid-sensing ion reduces and reduce cancer pain [16]. The bisphosphonate drugs should be considered as the treatment drugs when the analgesic drugs and radiation therapy are not effective in the treatment of bone cancer pain. These drugs are safe to use but have not proven as the most effective treatment model for alleviating pain due to cancer metastasis.

Tri-cyclic Antidepressants

Another drug used to treat bone pains in metastasis is tri-cyclic antidepressant (TCA) in cancer patients due to their positive effects on mood and sleep. The efficacy of these drugs for treating malignant pain is limited but its use in the treatment of nonmalignant pain is well studied and proved [17]. Various clinical trials and physicians have reported their effectiveness for changing the pain perception and reducing depressive symptoms in the cancer patients. So, their use can be justified as they have an antidepressant action that helps in advanced cancer patients. However, the use of TCA, especially in medically ill or elderly patients may be limited due to frequent side effects similar to those seen with opiates, which include drowsiness, constipation, urinary retention, and dry mouth, as well as such serious adverse effects as orthostatic hypotension, coma, liver function impairment, and cardiotoxicity [18]. But few selective serotonin reuptake inhibitors (SSRIs) such as paroxetine, citalopram, and selective norepinephrine reuptake inhibitors (SNRIs) such as venlafaxine and duloxetine have proved to be efficacious for treating neuropathic pain.

Corticosteroids

Corticosteroids which belong to another major group of medications are widely used as an adjuvant therapy for cancer-related pain syndromes. These are bone pain, neuropathic pain from infiltration or metastatic compression of neural structures, headache due to increased intracranial pressure, arthralgias, pain due to ongoing inflammation and the pressure on surrounding structures, and pain due to obstruction of hollow viscus or distention of an organ capsule [19]. However, it should be always taken into attention that corticosteroids, when used for a longer period of time, can produce significant adverse effects, such as immunosuppression, hypertension, hyperglycemia, gastric ulcers, and psychosis; although in cancer patients the risk versus benefit analysis reveals benefits that outweigh the risks involved in the use of steroids, particularly in cases of central nervous system involvement.

Growth Factors and Signaling Molecules

Another treatment modality caters the use of growth factors and signaling molecules responsible for the growth. One of them is osteoprotegerin which is a negative regulator of bone dissolving cells. It is among the class of TNF receptors. It inhibits bone destruction by activating the receptor activator of nuclear factor-kappa B ligand (RANKL) on osteoclasts and thus augment the apoptosis of osteoclasts [9]. This apoptosis causes the reduction in the amount of bone damage and thus reduces pain. This also helps in reducing the number of pathological fractures and the pain associated with them as well.

Endothelin-1 Receptor Antagonists

The endothelin-1 (ET-1) is a neurotransmitter that is secreted by neuronal cells, non-neuronal cells, and tumor cells [20]. Hyperalgesia in bony metastasis occurs due to sensitization of primary afferent nociceptors that contain ET-1 receptors. Therefore, ET-1 receptor antagonist causes alleviation of bone pain by antagonizing the effect of nociceptive stimuli at the receptors [21]. The ET system drugs, such as atrasentan, have been investigated for the clinical treatment of pain by causing the release of beta-endorphins and activation of the opioid pool. These antagonists have an indirect effect as well in which they cause a decrease in the disruption of cellular junctions preventing metastasis. The ET receptor antagonists may provide new advancement in the treatment modalities for bone pain in advanced carcinomas.

Radiotherapy

Radiotherapy (RT) is the most effective mode of treatment for alleviating pain in cancer patients. The Radiation Therapy Oncology Group reported that 80%–90% of patients receiving RT for osseous metastases experience partial to complete pain relief within 10-14 days of RT initiation [22]. Three types of radiotherapies are used for the treatment of bone metastases—one is external beam radiotherapy (EBRT), hemi-body irradiation (HBI), and radiopharmaceuticals [23]. Systematic review shows that EBRT, whether given as single or multiple fractions, produces 50% pain relief in 41% of patients and complete pain relief at one month in 24% of patients [24]. Also, a prospective study involving 91 patients with painful bone metastases who were treated with a median total dose of 46 Gray (Gy) found that complete and partial pain relief (≥50%) were obtained in 49% and 91% of patients, respectively [25].

There is no difference in the degree of pain relief depending on the fractions of RT. This is proved by the systemic review and meta-analysis of randomized controlled clinical trials which found that single-fraction RT with 1 × 8 Gy is as effective for pain relief as multi-fraction regimens such as 5 × 4 Gy in one week or 10 × 3 Gy in two weeks [26]. Although the optimal dose fractionation for radiation of metastatic bone lesions has been debated, an internet survey consisting of radiation oncologists, with members participating from the American Society for Radiology Oncology, Canadian Association of Radiation Oncology, and Royal Australian and New Zealand College of Radiologists, concluded that the most accepted fractionation schemes are 8 Gy in a single fraction and 30 Gy in 10 fractions [27].

Radioactive isotopes of phosphorus (P)-32 and strontium (Sr)-89 were the first bone-seeking radiopharmaceutical drugs approved by the United States (US) Food and Drug Administration (FDA) for the treatment of painful bone metastases, followed by samarium (Sm)-153, rhenium (Re)-186, and Re-188 [28]. Sr-89 chloride (Metastron™) and Sm-153–lexidronam (Quadramet®) are effective for treating ProstaticCa cell-induced bone metastases with 80% of patients having osteoblastic lesions achieving pain relief following strontium-89 administration [29-30]. In patients with metastatic bone pain, a Cochrane review found evidence to support their use as analgesics with a number needed to treat (NNT) of five and four to be complete and complete/partial relief, respectively [31-32]. Survival benefits have been shown by radium use in patients with castration-resistant prostate cancer. In Phase II clinical studies, the α-emitting radioisotope radium (Ra-223) demonstrated significant improvements in overall survival. Also, there was significant improvement seen in pain response as well as biochemical parameters [33]. However, Phase III randomized clinical trial (ALSYMPCA) aimed at the analysis of the analgesic efficacy, survival benefit as well as safety profile of Ra-223 (50 kBq/kg i.v.) is currently ongoing (NCT00699751).

Surgical management of metastatic bone pain 

Surgery is very rarely considered an option to treat bone pains due to metastatic lesions. This trend is not as popular as various pharmacological drugs over the years have gained success in achieving adequate pain control. Among them, long-acting opioids are effective for managing pain. Patients have reported pain relief with oxycodone, morphine, and fentanyl patches. Not only drugs but nerve blocks, neurolytic agents, and radiofrequency ablations have been getting immense popularity for the relief of this pain. Two types of surgical procedures have been used so far. One is neurodestruction and the other is neuromodulation.

Neurodestruction

Neurodestruction causes damage and disruption of the pain pathways carrying the signal through the spinal cord to the brain. This disruption can be done at any level, either it is nerve, nerve root, nerve root ganglia, spinal cord, thalamus or brain stem or in combination if the disease process is complex. The level of the block depends on the severity of pain. This procedure has been tried in many patients including those with spinal metastatic disease and has been found effective with long-lasting effects. Procedures like anterior decompression and spinal stabilization have proved to be effective without any progression in the neurological impairment making these procedures widely acceptable for use [34]. There are many procedures being used for this process but one of the most common is spinal cordotomy that disrupts the spinothalamic tract at the level of cervical or thoracic spinal cord. This causes loss of pain sensation from the opposite part of the body thus relieving pain [35]. This procedure causes insensitivity towards pain perception mimicking neuropathy of a particular site which forms the basis of its complications. It can cause accidental burns, ulcerations due to lack of sensations, dry damaged skin, and various others. Therefore midline myelotomy which is a form of the same procedure is reserved for only those patients who have visceral bilateral pain that is resistant to other modes of treatment. In midline myelotomy; the central spinal cord is disrupted but it involves a nonspecific pathway for the disruption of pain signal transmission [36]. Thalamotomy is another procedure which is done at the level of nuclei in somatosensory areas and anterior areas of thalamus. These areas relay pain, therefore, are used for malignant intractable pain. Contrast-guided (CT-guided) anteromedial pulvinotomy and centromedian thalamotomy are done in this procedure for pain relief [37]. Another neurodestructive procedure is cingulotomy that involves disruption of the pathway at the level of the limbic system which also modulates the psychological effects of pain and memory associated with the pain. But it is reserved for patients with resistant pain that has failed to respond to palliative pharmaceuticals because of the neurocognitive impairment the patients experience after the procedure. A case report was also published describing the effectiveness of this procedure citing three patients who underwent this treatment [38].

There are many benefits of neurodestructive procedures. The procedures are easy to perform with the modern medical technology, cause immediate pain relief, pain relief in resistant cases, and long-lasting effects as compared to pharmacological drugs. However, these procedures are irreversible, cause numbness, weakness, paresthesia, neurocognitive impairment, and inability to use for future testation regarding the effectiveness of the treatment. The numbness and weakness take a long time to recover during which patient is at higher risk for the development of other complications, especially with bilateral procedures. Also, there are certain limitations of the procedures. They are contraindicated in coagulopathies (which is common in most visceral cancers due to the release of substances that causes hypercoagulable states of the body). These procedures are particularly useful if the life expectancy is two to three months because their effect lasts for three to four months. These procedures will render the patient free of any drug use for pain control later on thus decreasing the burden of adverse effects of pharmacological therapy. Injections of neurolytic substances at the ganglion are also an effective mode of treatment for treating pain. We can treat chronic abdominal pain associated with pancreatic cancer by celiac plexus block (injection of a neurolytic agent near the celiac plexus at the level of T-12). This block has proved to be effective and safe to control pain by providing painlessness in 70%-90% of patients with various types of abdominal cancers with mean pain decreased by 40% in the majority of patients [39]. Celiac plexus block causes orthostatic hypotension, local pain, and diarrhea as the most common side-effects, but can be managed with early detection and adequate conservative treatment. In other cases, hypogastric plexus block can also be used. It is used in cases of visceral and pelvic pain associated with extensive gynecologic, colorectal, or genitourinary cancers [40]. However hypogastric plexus block is rarely used as it is found to be less effective than celiac plexus block because of the widespread extent of the disease at the time of diagnosis in this group. But in cases of medically intractable pelvic pain, a hypogastric block can still be used and it has no serious complications reported till far. Local nerve blocks or neurolysis with phenol or alcohol can also be used for treating localized pain and kyphoplasty can be used for painful vertebral compression fractures in patients with metastatic cancer [41-42].

Neuromodulation

Electrical neuromodulation is the second surgical mode of treating pain in patients. This procedure involves the electrical stimulation of peripheral nerve or dorsal column of spinal cord and brain. Spinal cord stimulation primarily deals with neuropathic pain, such as in patients with arachnoiditis, but it has not played a significant role in the perception of nociceptive pain. Spinal cord stimulation causes 60% reduction in the severity of pain which has improved the quality of life for three years or more but it is not yet considered as one of the first line treatment options for intractable pain [41].

Another mode of treating cancer pain is the use of drugs intrathecally that reduce the perception of pain. For this various drugs can be used including opioids, ziconotide, local anesthetics, and baclofen. Intrathecal opioids given alone or in combination with other drugs such as alpha agonists or local anesthetics are used for intractable pain relief. These drugs are mostly given by self-controlled pump that delivers the medication at a specific rate in the intrathecal space depending on the requirement and severity of pain. Intrathecal administration of these drugs helps reduce systemic side effects of the drugs and thus are widely accepted in those with contraindications of these drugs due to co-morbid conditions. This also helps in increased amounts of cerebrospinal fluid (CSF) concentrations of the drug that would increase the sensitivity of the drug and would require lesser dosages to alleviate pain.

Intrathecal drug delivery system leads to comprehensive management of cancer pain. This is proved by one multicenter, randomized clinical trial which demonstrated that patients with refractory cancer pain are more effectively treated with the addition of an implantable intrathecal drug delivery system to the standard therapy [43]. The systemic side effects were decreased by 50% through infusion by the intrathecal pump. Patients reported lesser fatigue, sedation, constipation and also showed improved survival rate at six months. And finally, patients with the implanted intrathecal drug delivery system had significant reduction of fatigue and depressed consciousness, as well as an improved rate of survival at six months [44]. This pump is implanted in the subcutaneous fat of the abdomen that provides continuous infusion. More commonly used pumps nowadays have programmable devices that contain an electronic module that allows adjustment of the drug infusion rate using telemetry programming. All pumps have to be refilled at regular time intervals which are done every one to three months in office or clinic settings by simple insertion of the needle into the center of the reservoir through the skin. Also, clonidine and bupivacaine are the most commonly used non-opioid medications for intrathecal administration in cancer patients. They are both used in combination with morphine to strengthen its analgesic effect. Clonidine produces analgesia by its action on alpha-2 receptors on presynaptic primary afferents and postsynaptic dorsal horn neurons of the spinal cord and causes a decrease in the release of neurotransmitter from C-fibers (e.g., substance P) and thus cause inhibition of preganglionic sympathetic transmission [45]. Local anesthetic bupivacaine can also be used to produce its analgesic effect by blocking voltage-sensitive sodium channels. This prevents the generation and conduction of nerve impulses. But we have limited its use due to side effects including neuropathy, cardiotoxicity, and bladder and bowel incontinence. These adverse effects can be controlled by slow titration.

GABA-B agonist baclofen can be used in cancer patients who experience severe spasticity [46]. When administered intrathecally, baclofen inhibits both monosynaptic and postsynaptic reflexes at the spinal level and help restore electrical signals that cause relaxation of the muscles. Baclofen is used for treating neuropathic pain but it has various side effects as well including sedation, hypotonia with weakness, and urinary retention. Sudden discontinuation of the therapy can be life-threatening as severe rebound spasticity occurs that leads to intractable pain. It is also accompanied by high fevers, confusion, muscle weakness, rhabdomyolysis, and multiple organ failure.

Several years ago Elan Pharmaceuticals introduced a new analgesic drug, ziconotide, which is a synthetic, non-opioid analgesic agent for the amelioration of severe and chronic pain. It was given the FDA approval in 2004 after several human and animal safety and efficacy studies. Ziconotide binds to specific N-type voltage-sensitive calcium channels found in neural tissue and acts by inhibiting the release of nociceptive neurochemicals like substance-P, glutamate, and calcitonin gene-related peptide in the spinal cord, thus relieving pain [47]. Although it is a safe drug as the long-term side effects are unknown and also due to severe adverse effects during the initial phase of starting the therapy, it is not considered the first choice for many patients for pain management [48].

The adjustability and reversibility of intrathecal pumps provide added benefit for controlling the rate of infusion and drug composition during the course of therapy. Also, this mode of therapy is highly testable as the patients provide data on the degree of pain relief and the effectiveness of the treatment mode. This mode of treatment carries various risks and side effects along with it such as site infection, increased costs, long term treatment, and device malfunctions. Besides adverse effects, there are not many serious complications reported for intrathecal drug delivery system implantation itself. Some of the common complications are infection of the meninges, granuloma formation at the tip of the subarachnoid catheter, bleeding or hematoma at the site of the surgery, and malfunctioning of the device. Theses device malfunctions are reversible and the rest of the complications are treatable. This makes the use of intrathecal pumps as a widely accepted mode of treatment for long-term cancer patients suffering intractable pain due to bony metastasis [49].

## Conclusions

Keeping in mind all the medical and surgical options available, it can be safely concluded that pain management is extremely crucial in cancer patients. Even though we have a multitude of options for this purpose, we are still far from pain-free outcomes. The future prospects of metastatic pain management are still wide open to further enhance patient satisfaction, decrease psychological impacts, and improve the overall quality of life of the patient.
